# Nanoparticle Reinforced Polymers

**DOI:** 10.3390/polym11040625

**Published:** 2019-04-04

**Authors:** Ana María Díez-Pascual

**Affiliations:** Department of Analytical Chemistry, Physical Chemistry and Chemical Engineering, Faculty of Sciences, Institute of Chemistry Research “Andrés M. del Río” (IQAR), University of Alcalá, Ctra. Madrid-Barcelona, Km. 33.6, 28871 Madrid, Spain; am.diez@uah.es; Tel.: +34-918-856-430

The beginning of nanomaterials and nanoscience dates back to 1959 when the Nobel laureate in Physics Richard Feynman gave the famous lecture entitled “There’s Plenty of Room at the Bottom.” Nonetheless, the concept of nanoparticle-reinforced polymers came about three decades ago after the outstanding discovery of fullerenes and carbon nanotubes. The addition of these nanoparticles into different polymer matrices opened up an important research area in the field of composite materials. Furthermore, many different types of inorganic nanoparticles, such as quantum dots, metal oxides, and ceramic and metallic nanoparticles, have been incorporated into polymers for application in a wide range of fields, such as medicine, photovoltaics, packaging and structural applications, etc. One of the main ideas behind this approach is to improve the matrix mechanical performance. The nanoparticles exhibit higher specific surface area, surface energy, and density compared to microparticles, hence lower nanofiller concentrations are needed to attain properties comparable to or even better than those obtained by conventional microfiller loadings, which facilitate processing and minimizes the increase in composite weight. Nonetheless, a number of challenges have to be faced. Nanoparticle agglomeration within the polymer matrix and poor interfacial adhesion between the nanofillers and the matrix frequently hinder property improvements, demanding modification of the surface chemistry to promote physical or chemical interactions with the polymer chains. Furthermore, the fabrication procedure of the nanoparticle-modified polymers leads to different morphologies that have a strong influence on the final properties of the materials. This Special Issue, with a collection of 12 original contributions and four reviews, provides selected examples of the most recent advances in the preparation, characterization, and application of polymeric nanocomposites comprising nanoparticles. 

Recently, inorganic fullerenes, nanotubes, and 2D nanomaterials based on layered metal dichalcogenides such as WS_2_ and MoS_2_, have emerged as being among the most promising polymer reinforcements. These types of nanoparticles are currently the subject of intense research, summarized in different literature reviews [1,2,3]. In particular, the incorporation of environmentally-friendly tungsten disulfide (WS_2_) nanoparticles has been shown to improve the thermal, mechanical, and tribological properties of a series of thermoplastic polymers, including isotactic polypropylene (iPP) [4], polyphenylene sulfide (PPS) [5], and poly(ether ether ketone) (PEEK) [6]. Efficient dispersion of WS_2_ was achieved through simple melt-blending without using modifiers or surfactants. Continuing progress in the field, WS_2_-based nanotubes have been used to prepare poly(3-hydroxybutyrate-co-3-hydroxyvalerate) (PHBV) nanocomposites by blending in solution [7], and the effects of the nanotubes on the isothermal crystallization behavior and kinetics of PHBV were investigated by differential scanning calorimetry (DSC) and polarized optical microscopy (POM). Results revealed that very low nanotube loadings (0.1–1.0 wt %) raised the crystallization rate of the matrix due to the heterogeneous nucleation effect and provided understanding of the structure-property relationships in PHBV-based nanocomposites that are useful for their practical applications. Hybrids comprising reduced graphene oxide (rGO) and WS_2_ nanoparticles grafted with active amino groups were used to develop bismaleimide (BMI) nanocomposites (NH_2_-rGO/WS_2_/BMI) via the casting method [8]. Results demonstrated unprecedented enhancements in the mechanical properties of the BMI resin with trace amounts of nanofillers. In particular, only 0.6 wt % NH_2_-rGO/WS_2_ increased the impact and flexural strength of the matrix by 91.3% and 62.6%, respectively, ascribed to the layered structure of the hybrid nanoparticles, strong interfacial adhesion, and uniform dispersion of the NH_2_-rGO/WS_2_ within the resin. Besides, the thermal stability significantly improved with the addition of the nanoparticles. 

Monolayers of molybdenum disulfide (MoS_2_) are of great interest to tailor the mechanical and gas barrier properties of polymeric materials. In this regard, Tsai et al. [9] exfoliated MoS_2_ via modification with ethanethiol and nonanethiol to obtain monolayers with thicknesses in the range of 0.7–1.1 nm, which were then added to chlorobutyl rubber to prepare MoS_2_-butyl rubber nanocomposites with loadings of 0.5, 1, 3, and 5 wt %. The tensile stress and oxygen transmission rate of the matrix showed maximum augments of 30.7% and 49.6%, respectively, making these nanocomposites highly suitable to be applied as pharmaceutical stoppers. Another field of application of rubber-based nanocomposites is tire manufacturing. Thus, the development of silica/rubber composites with low rolling resistance in an energy-saving method is of great interest. Zheng and coworkers [10] proposed a novel strategy in which alcohol polyoxyethylene ether (AEO) and a silane coupling agent (K-MEPTS) were grafted onto the surface of silica nanoparticles and subsequently incorporated into a rubber matrix using the latex compounding technique. The characteristics of the resulting nanocomposites were studied by a rubber process analyzer (RPA), transmission electron microscopy (TEM), and tensile tests. Results revealed that AEO forms a physical interface between the nanoparticles and the matrix that reduces silica aggregation, while K-MEPTS forms a chemical interface that enhances the reinforcing effect of the nanoparticles and minimizes the joint friction between them. Overall, AEO and K-MEPTS acted synergistically to improve the mechanical and dynamic performances of silica/rubber nanocomposites. 

Noble metal nanoparticles (NPs) are very attractive catalysts due to their highly active surface atoms [11]. Nonetheless, owed to their high surface-to-volume ratio and surface energy, they can easily agglomerate during the catalytic reaction process. To overcome this issue, NPs are commonly stabilized by polymers, complex ligands, or surfactants [12], albeit the catalytic reactivity of the resulting blends is low. To solve this drawback, Wang and coworkers [13] developed a composite membrane with SiO_2_ microspheres coated by Ag NPs on a PVDF substrate. The membrane displayed high catalytic reactivity when catalyzing the reduction of p-nitrophenol to p-aminophenol, an important fine chemical intermediate, under a cross-flow. The reaction followed a first-order kinetics, and its speed raised with increasing the Ag concentration in the membrane, the reaction temperature, and the operating pressure. Therefore, the developed reaction system will be of great interest for the chemical industry. 

On the other hand, AuNPs can be used as colorimetric sensors for Ag+ detection. However, AuNPs-based sensors generally suffer from poor selectivity and low sensitivity, owed to the nanoparticle tendency to aggregate during their synthesis and sensing process. To address this issue, Liu et al. [14] functionalized the nanoparticles with a thermoresponsive polymer that acted as both a reducing and stabilizing agent: hyperbranched polyethylenimine-terminal isobutyramide (HPEI-IBAm). The modified nanoparticles showed outstanding thermal stability up to 200 °C, high tolerance of a wide range of pH, and high salt resistance. The developed colorimetric sensor exhibited high stability, selectivity, and sensitivity, with an extremely low detection limit of 7.22 nM. More importantly, due to the thermoresponsivity of HPEI-IBAm, the AuNPs/Ag composites could be separated by heating the system above the cloud point of the polymer solution followed by centrifugation, thereby preventing and controlling Ag+ pollution to the environment.

AuNPs have also been successfully used for the colorimetric detection of melamine (C_3_H_6_N_6_), a white heterocycle compound extensively applied in the manufacturing of plastics, laminates, dyes, and fertilizers that can cause health problems or even death [15]. However, the current methods for melamine detection involve time-consuming steps, complicated procedures, and expensive equipments. Zhang et al. [16] developed a fluorescence resonance energy transfer (FRET) system for melamine detection based on AuNPs and conjugated polymer nanoparticles (CPNs), which led to a maximum energy transfer efficiency of 82.1%. The system was found to be highly selective and sensitive to melamine detection, with a detection limit of 1.7 nmol/L. Results revealed that the fluorescence of CPNs was initially quenched by AuNPs and then restored after melamine addition since it reduced the FRET between CPNs and AuNPs. Moreover, the proposed method was tested in powdered infant formula with very good results. A FRET mechanism was also designed for ratiometric fluorescent temperature sensing based on two thermo-responsive polymers, poly(*N*-isopropylacrylamide) and poly(N-isopropylmethacrylamide), which present lower critical solution temperatures (LCSTs) of 31.1 and 48.6 °C, respectively [17]. The fluorescent intensity of each polymer (with maxima at 436 and 628 nm, respectively) decreased upon heating the polymer aqueous solution. When the two polymer aqueous solutions were mixed, the fluorescent intensity at 436 nm decreased while that at 628 nm increased with increasing temperature owed to the FRET effect. This temperature sensing system displayed good stability and biocompatibility, and could be very interesting for biomedical applications, such as bioimaging of biological processes at specific organelles. 

The FRET mechanism also influences the power conversion efficiency in polymeric organic light-emitting diodes (OLEDs). The energy emission may be reduced or even eliminated when the nonradiative energy transfer processes is very efficient [18]. This drawback can be addressed by the incorporation of nanostructured materials such as SiO_2_/TiO_2_ nanocomposites. Thus, the performance of OLEDs based on hybrids of poly(9,9-di-*n*-octylfluorenyl-2,7-diyl) (PFO), which acts as a donor material, and poly(2-methoxy-5-(2-ethyl-hexyloxy)-1,4-phenylene-vinylene) (MEH-PPV), which acts as an acceptor material, was significantly improved upon addition of SiO_2_/TiO_2_ nanoparticle blends [19]. An improved charge carrier injection and an important reduction in the turn-on voltage of the devices was observed in the presence of the nanocomposites, as well as an enhancement of the MEH-PPV electroluminescence peaks, not only due to the reduction of the FRET effect but also to a hole-electron recombination. 

It should be noted that SiO_2_ and TiO_2_ are among the most common inorganic oxide nanofillers used in polymer-based nanocomposites. An effective approach to achieve their homogeneous dispersion within the matrix is in situ synthesis using techniques such as the sol–gel, microemulsion, or hydrothermal/solvothermal methods [20]. These methods typically involve the mixing of precursors with a non-reactive solvent and the monomer, and the reaction of the precursors starts the nanoparticle synthesis either before or during monomer polymerization. This bottom–up approach allows better control over the structure and properties of the nanocomposites; given that the nanoparticles are grown inside the polymer matrix, the passivating effect of the polymer chain functional groups on the nanoparticles can control particle size and decrease agglomeration. However, the unreacted precursors or byproducts of the in situ reactions frequently modify the properties of the nanocomposite. Considering the large number of studies published on the in situ synthesis of SiO_2_ and TiO_2_ nanofillers, Adnan et al. [21] collected the most relevant results in the field, focusing on the sol-gel method and thermoset matrices like epoxy resins and poly(dimethylsiloxane) (PDMS). In addition to the preparation techniques and strategies, the properties and most relevant applications of such nanocomposites are discussed, including laminates, structural composites, electrical insulation, coatings for epoxy and optical devices, and coatings and bioactive materials for PDMS.

Carbon based nanoparticles such as graphite carbon nanoparticles (CNPs) have also been used as reinforcing materials of different matrices including epoxy resins. In this regard, Nezhad and Thakur [22] reported on the preparation and mechanical characterization of epoxy/CNPs nanocomposites with nanoparticle loadings in the range of 1–5 wt%. Embedment conditions and timing were optimized, and the failure strain, hardness, strength, and modulus of the different samples were measured. A worsening in properties was found upon addition of the highest CNPs loading, attributed to a morphological change, including re-agglomeration of the nanoparticles. On the other hand, graphene and its derivatives, graphene oxide (GO) and reduced graphene oxide (rGO), have huge potential for energy applications due to their 2D structure, large specific surface area, high thermal conductivity, optical transparency, and enormous mechanical strength combined with intrinsic flexibility. The combination of graphene-based nanomaterials with polymers opens up an interesting research field, leading to novel nanocomposites with improved structural and functional properties due to synergistic effects. In this regard, a brief review [23] that summarizes the most recent progress in the development of graphene/polymer nanocomposites for use in polymer solar cells is timely. These nanocomposites have already been used as transparent conducting electrodes, active layers, and interfacial layers of such type of cells. Characteristic photovoltaic parameters including the open-circuit voltage, short-circuit current density, fill factor, and power-conversion efficiency are comparatively described for the different device structures, and future perspectives in the field are discussed.

Incorporating a small amount of nanofillers into polymers is an effective approach to enhance their electrical breakdown strength [24]. However, nanoparticle surface modification and/or chemical grafting to the matrix are generally required to attain strong nanofiller-matrix interfacial adhesion and homogeneous nanoparticle dispersion. Accordingly, surface modified MgO, TiO_2_, Al_2_O_3_, and ZnO nanoparticles have been modified by a silane coupling agent and then incorporated into dielectric polymers such as polypropylene (PP), polyester, and polyimide (PI) to fabricate nanocomposite dielectric materials [25]. In this regard, Min et al. [26] fabricated PP/Al_2_O_3_ nanocomposites with silane-modified Al_2_O_3_ contents in the range of 0.5–2 wt % via ultrasonication followed by melt blending, and their trap distribution and dc electrical breakdown properties were measured. It was found that the nanocomposites with the lowest loading displayed the best electrical breakdown strength, attributed to the formation of isolated interfacial regions that reduced the effective carrier mobility and strengthened the interaction between molecular chains, thereby hindering the charge transport and the displacement of molecular chains with occupied deep traps. Nanocomposites based on other commodity thermoplastic polymers such as polyethylene congeners (i.e., LDPE, HDPE, and XLPE) are widely used as dielectric materials, in particular for power cable insulation. Continuing progress in this topic, Pleşa et al. [27] reviewed the structure-property relationships of these nanocomposites and their electrical performance. In particular, their electrical conductivity, relative permittivity, dielectric losses, partial discharges, space charge, electrical and water tree resistance behavior, and electric breakdown are discussed in detail.

Besides the investigation on the nanoparticle dispersion and nanofiller-matrix interfacial adhesion, other parameters such as the polymer conformation, packing, and dynamics of adsorption at the matrix-nanoparticle interface have been investigated by several authors [28,29]. Continuing progress in this topic, Song et al. [30] analyzed the effect of the polymer chain topology structure on the adsorption behavior at the polymer-nanoparticle interface by coarse-grained molecular dynamics simulations. For weak polymer-nanoparticle interactions, ring chains with a closed topology structure prevail at the interfacial region, whereas for strong interactions, linear chain preferential adsorption takes place. These results are interesting for the design of high-performance nanocomposites with tailored morphology. 

Another current interesting topic is the incorporation of nanoparticles into hydrogels, which leads to multifunctional nanocomposite polymers widely used in a number of applications, ranging from drug delivery to bioimaging and tissue engineering. Dannert et al. [31] reviewed different reported methods to integrate nanoparticles into hydrogels, the interactions in these composites, and how these support the enhanced mechanical and optical properties of the hydrogels, including their self-healing ability. Furthermore, the ability of the nanofillers to tailor the equilibrium swelling and elasticity of the hydrogels is discussed, as well as the use of this type of polymers as dispersing agents for nanomaterials, consequently enabling an improved performance in catalytic and sensor applications. 
